# Opposite effects of rapid auditory stimulation on tetanized and non-tetanized tone of adjacent frequency: Mismatch negativity study

**DOI:** 10.1371/journal.pone.0289964

**Published:** 2023-08-11

**Authors:** Daria Kostanian, Daria Kleeva, Gurgen Soghoyan, Anna Rebreikina, Olga Sysoeva

**Affiliations:** 1 Center for Cognitive Sciences, Sirius University of Science and Technology, Sochi, Russia; 2 Center for Bioelectric Interfaces, National Research University “Higher School of Economics”, Moscow, Russia; 3 V. Zelman Center for Neurobiology and Brain Restoration, Skolkovo Institute of Science and Technology, Moscow, Russia; 4 Laboratory of Human Higher Nervous Activity, Institute of Higher Nervous Activity and Neurophysiology of RAS, Moscow, Russia; Stanford University School of Medicine, UNITED STATES

## Abstract

Our study describes the effects of sensory tetanization on neurophysiological and behavioral measures in humans linking cellular studies of long-term potentiation with high-level brain processes. Rapid (every 75ms) presentation of pure tone (1020 Hz, 50ms) for 2 minutes was preceded and followed by oddball blocks that contained the same stimulus presented as deviant (probability of 5–10%) interspersed with standard (80–90%) and deviant tones (5–10%) of adjacent frequencies (1000 and 980Hz, respectively). Mismatch negativity (MMN) component in response to tetanized tone (1020Hz), while being similar to MMN for non-tetanized tone before tetanization, became larger than that after tetanization, pointing to the increase in cortical differentiation of these tones. However, this differentiation was partially due to the MMN decrease after tetanization for tones adjacent to tetanized frequency, suggesting the influence of lateral inhibition to this effect. Although MMN correlated with tone discriminability in a psychophysical task, the behavioral improvement after tetanization was not statistically detectable. To conclude, short-term auditory tetanization affects cortical representation of tones that are not limited to the tetanized stimuli.

## Introduction

The plastic changes, resulting from exposure to various stimuli, are the basis of learning and memory, which help adapting to a changing environment. Sensory-dependent changes in brain activity have been shown at different levels of information processing in both invasive animal studies (e.g. [1–3]) and non-invasive human studies (e.g. [4–9]). A relatively new paradigm that uses rapid sensory stimulation/tetanization is of particular interest as it bridges human and animal studies. Such rapid (around 10–20 Hz) and short-term (about 2 minutes) stimulation in humans both in visual or auditory modality is proposed to cause long-term potentiation (LTP) like effects similar to those caused by electrical tetanization previously rigorously studied at the cellular level in animals [4, 10–12]. LTP is defined as the strengthening of synapses under the influence of repetitive stimulation. This strengthening of connections is mediated by a change in the activation of postsynaptic N-methyl-D-aspartate receptors (NMDAR) [13] and an increase in the density of α-amino-3-hydroxy-5-methyl-4-isoxazolepropionic acid receptors (AMPAR) on the surface of the postsynaptic membrane [14, 15]. Similar changes in LTP effects with NMDAR agonists/antagonists have been found both after sensory [16] and electrical tetanization in vivo [3, 17] and after electrical tetanization in vitro [18]. These data provide evidence that rapid and short-term sensory stimulation can induce long-term potentiation.

Numerous studies suggest that event related potentials (ERP) can be used as a marker of neurophysiological changes associated with sensory experience and auditory discrimination training. For example, training to discriminate different properties of auditory stimuli leads to the increase of ERPs components such as N1, P2, and mismatch negativity (MMN) [19–23]. Sensory tetanization effects on the components of evoked potentials may be an approach to move from the cellular to the systemic description of neuroplastic changes related to LTP (kind of long-term adaptation of a neural system), as both visual and auditory tetanization has also been shown to affect these ERP components. At the same time results are rather inconsistent, probably related to different experimental designs across studies [4–6, 12, 24–27]. Moreover, the recent meta-analysis [28] revealed that the overall effect of tetanization across ERP studies is not significant. However this meta-analysis pointed to the relatively few studies on auditory tetanization that might be of importance due to potential link to speech development. The effects of auditory tetanization on MMN have been studied in only two studies[5, 29], providing discrepant results, thus calling for additional research.

MMN response is obtained as a differential neurophysiological response to standard (frequent) and deviant (rare) stimuli presented in an odd-ball paradigm. MMN amplitude has been shown to be related to behavioral discrimination ability despite it is typically recorded in a passive paradigm with auditory stimuli presented at the background when participants are engaged in the watching muted videos, [30]. Kompus and Westerhausen [5] have reported MMN increase after tetanization of 13 Hz, while Rebreikina and colleagues [29] have not revealed this effect after 26 Hz tetanisation. In Kompus and Westerhausen the MMN enhancement has been specific to the deviant stimuli have been presented during tetanization (1025 Hz) but not to the tone of neighboring frequency (975 Hz), suggesting subtle resolution of tetanization effects [5]. As MMN is believed to reflect the subjective ability of an individual to differentiate the stimuli [30], the MMN increase has been interpreted as an indication of better discriminability of tetanized tones, however no behavioral assessment was performed [5]. As a field of cognitive neuroscience suffers with a low reproducibility of the results [31, 32] our current study aims to examine this MMN result further with slight modification of the experimental paradigms, as well as to relate these neurophysiological changes with the ability to discriminate sounds in behavioral tasks that was not done in previous research.

## Materials and methods

### Participants

Twenty-seven healthy young adults (11 males, mean age 23.3 ± 5.6) took part in the study. All participants completed a pre-study screening questionnaire. All participants did not report any neurological, psychiatric or hearing disease, brain injuries and had not taken any medicine in the six months before the study. Also, participants completed a functional state questionnaire on the day of the study and reported that they had not consumed alcohol, caffeine or nicotine before the experiment. Three of the 27 participants were left-handed. They participated in one of three versions of experiment with slightly varied parameters: 10 participants (8 males, mean age 22.9 ± 4.0) - in the Version 1; 10 participants (1 males, mean age 23.9 ± 2.26) - in the Version 2; and 7 participants (2 males, mean age 25.5 ± 3.65) - in the Version 3. Versions’ differences are provided in the procedure section of this manuscript. The data of one participant (from version 3) were lost due to technical issues. One participant (from version 2) was excluded from the sample because of an atypical EEG pattern. Two participants’ behavioral data were lost due to technical issues. 23 participants had both EEG and behavioral data.

The participants were recruited through the advertisements at the educational events being held by our organization. Before the experimental procedure, the nature of the study was explained to the participants. They were allowed to withdraw from the experiment at any time. The participants signed informed consent after the nature of the study was explained to them. At the end of the experiment, they received monetary gratification (500 rubles). The study protocol was approved by the Ethics Committee and met the standards for research from the Helsinki Declaration of 1975 (protocol 1 from 01.15.2020).

### Procedure

The stimuli were sinusoidal tones of three frequencies: 1020 Hz, 1000 Hz and 980 Hz, similar to those used in the experimental procedure in the Kompus and Westerhausen study [5]. The duration of each tone was 50 ms, the loudness was at 75 dB. The interstimulus interval was 400 ms. The stimuli were presented binaurally through earphones. Standard tones of 1000 Hz were interspersed with two deviants of 1020 Hz and 980 Hz. These particular frequencies were used as they are at the boundary of perceptual differentiation [21, 33]. The experimental blocks had three versions with slight modifications in the experimental design exploring the possibility to shorten the original version of this experiment for potential application into the clinical group. In the Version 1 each deviant was presented 150 times and the probability was 5% each as was originally set in Kompus and Westerhausen [5]. The total duration of this version was about 20 minutes. In Version 2 deviants number was decreased to 74 each. In the Version 3, each deviant tone was presented 150 times, but the deviance probability was increased to 10%. Thus, Version 2 and 3 lasted about 10 minutes and were equated with Version 1 by the deviance probability and number of stimuli, respectively. Both parameters influence the strength of MMN [34] thus potentially these experimental modifications might show differential sensitivity to tetanization effect. There was one block of the MMN sequences presented before and one block of the MMN sequences presented after tetanization. LTP-like stimulation (tetanization) lasted 2 minutes and consisted of the tetanized tone of 1020 Hz presented every 75 ms (roughly corresponding to 13.3 Hz frequency). In Version 2 and 3 the MMN blocks were separated from tetanization by 1-minute silent interval. In Version 1, in addition to MMN blocks, there were blocks of 1020 and 980 Hz sounds presented roughly with 2 seconds intervals before and after tetanization with a total duration of about 15 minutes. Results of these blocks are reported in a different paper. This version examines if the tetanization effect will persist for at least 15 minutes after the stimulation. During the sound presentation, participants watched self-selected muted movies (video).

Psychophysical blocks (about 5–10 min) that aim to estimate individual ability to discriminate the tones were presented twice: (1) before the tetanization block (but after the first odd-ball block) and (2) after the tetanization block and second odd-ball block at the very end of the study. In these psychophysical blocks we collected the behavioral data on the detection of subjective differences within the pairs of presented tones (two standard 1000 Hz, standard 1000 Hz and deviant 980 Hz, or standard 1000 Hz and deviant 1020 Hz). Each pair presented 5 times. After the presentation of each pair, the participants were asked whether the tones are the same or different. For each participant we calculated the number of hits (correctly detected differences), the number of false alarms (falsely detected differences in cases when the tones were the same), the number of misses (falsely rejected differences in cases when the tones were different), and the number of correct rejections (the cases in which the absence of differences was correctly reported). The obtained values were then analyzed within the framework of signal detection theory. We parameterized the sensitivity with the aprime - a non-parametric version of the more commonly used dprime measure, which reflects the interval between the signal and signal+noise distributions [35]. A’ (aprime) was computed using the following formula:

$$A^{\prime }\;=\;1\;-\;\frac{1}{4}\;*\;\left(\frac{FAR}{HR}\;+\;\frac{1\;-\;HR}{1\;-\;FAR}\right)$$

Where HR stands for hit rate (the percentage of correctly detected differences), FAR stands for false alarm rate (the percentage of falsely detected differences) and A’ or aprime stands for the sensitivity of the participant to tone discrimination. Aprime ranges from 0 to 1, where 0.5 is chance level and 1 is maximum sensitivity (perfect score). Corresponding toolbox in ‘psycho’ [36] package in R was used.

### EEG recording

EEG signals were recorded in a dimly lit soundproof room using 128-channel caps (EasyCap). EEG signal was recorded continuously with 500 Hz sampling rate and 140 Hz lowpass filtering with actiCHamp Plus amplifiers (Brain Products GmbH). Electrode impedances were kept below 15 kOm. A reference to the FCz channel was used during the EEG acquisition.

### EEG analysis

EEG processing was performed in MNE Python software [37]. EEG was FIR-filtered in the 0.5–20 Hz range twice - once forward and once backward (’zero-double’ phase in MNE Python toolbox). Based on their spectral characteristics, noisy/flat channels were interpolated. The data was divided into epochs from 100 ms before the stimulus onset and 450 ms after it. The epochs were rejected if the peak-to-peak signal amplitude exceeded 350 μV. Eye-movement correction was made by the means of ICA-decomposition and rejection of components corresponding to the horizontal and to the vertical eye movements, in particular the ALICE platform was used [38]. Before averaging, we dropped the epochs where the amplitude exceeded seven standard deviations of the mean over the whole data for each participant. The resulting minimal number of epochs was 70% from the original size of the set (mean number of trials averaged for each of the deviants was 107 ± 33, for standards - 1611 ± 663), thus allowing for further meaningful processing of ERPs. The epochs were averaged separately per each stimulus type and condition with the baseline within 100 ms before the stimulus onset. Data was re-referenced to the average over all electrodes.

The amplitude of MMN was estimated from the difference wave calculated by subtracting the evoked responses to the standard tones (1000 Hz) from those to the deviant ones (980 Hz or 1020 Hz). MMN amplitudes were calculated from difference waves as an average value over 100–250 ms post-stimulus period, the latency typically used for MMN calculation [5, 39].

### Statistical analysis

The repeated-measures analysis of variances (ANOVA) were applied to examine significant differences across Stimuli Type (tetanized 1020 Hz vs non-tetanized 980 Hz) and Tetanization (before vs after) for each electrode. As there were slight modifications in our experimental design that might influence the ERPs, our two main within-group factors, Stimuli Type and Tetanization, were supplemented by an additional between-subject factor - Version with three levels: Version 1, Version 2 and Version 3. This ANOVA analysis was applied to the amplitude of the MMN component. We looked at the effect of Tetanization as well as Tetanization by Stimuli Type interaction. Addressing the multiple comparison problem, we considered clusters as significant, if they consisted of at least 4 neighboring electrodes with significant differences. Assuming independence of our measures at each electrode, with α set to 0.05, the probability of a false alarm for this cluster was 4*0.05 = 6,25 * 10−6. Pearson correlation was used to examine interrelation between measures of interests.

## Results

### Neurophysiological (MMN) effects

**Fig 1:**
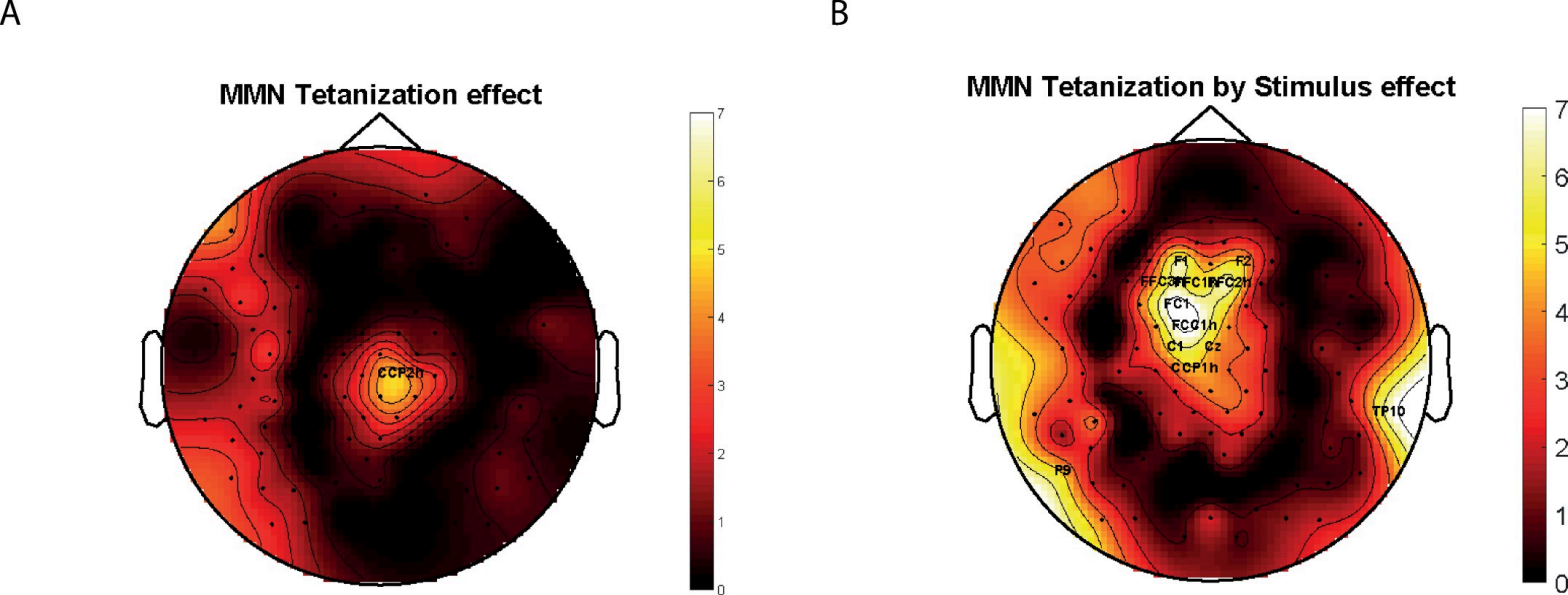
F-scores topography for tetanization and tetanization by Stimuli Type effects.

**Fig 2:**
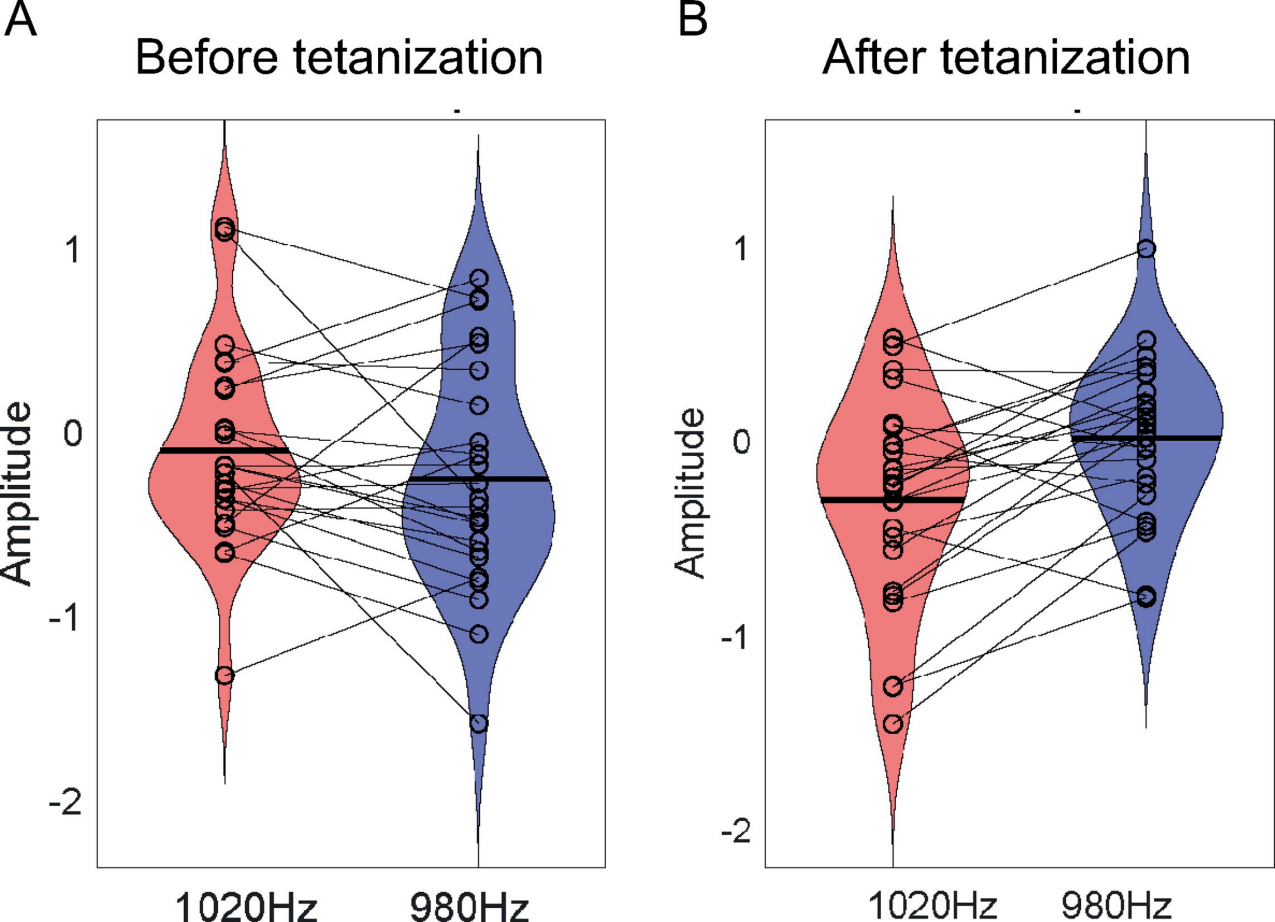
Violin plots representing distributions of MMN amplitudes A) before tetanization and B) after tetanization for tetanized deviant (1020 Hz)(red) and non-tetanized deviant (980 Hz) (blue).

Fig 1 represents the topography of the F-scores obtained in whole-channels ANOVA for the main effect of Tetanization and Tetanization by Stimuli Type interaction. The main effect of Tetanization was significant only at one electrode (CPz) and thus no significant clusters were formed. The Tetanization by Stimuli Type interaction were significant at ’F1’, ’F2’, ’FFC3h’, ’FFC1h’, ’FFC2h’, ’FC1’, ’FCC1h’, ’C1’, ’Cz’, ’CCP1h’ channels that form significant frontal-central cluster (averaged over the whole cluster statistics (F(1,24) = 5.7730; p = 0.029, eta2 = 0.194). Post-hoc follow-up analysis revealed that MMN in response to tetanized tone of 1020 Hz was not different from MMN to non-tetanized tone of 980 Hz before tetanization (p = 0.175) but became significantly larger than MMN for the non-tetanized stimuli after tetanization (p = 0.002) (Fig 2).

**Fig 3:**
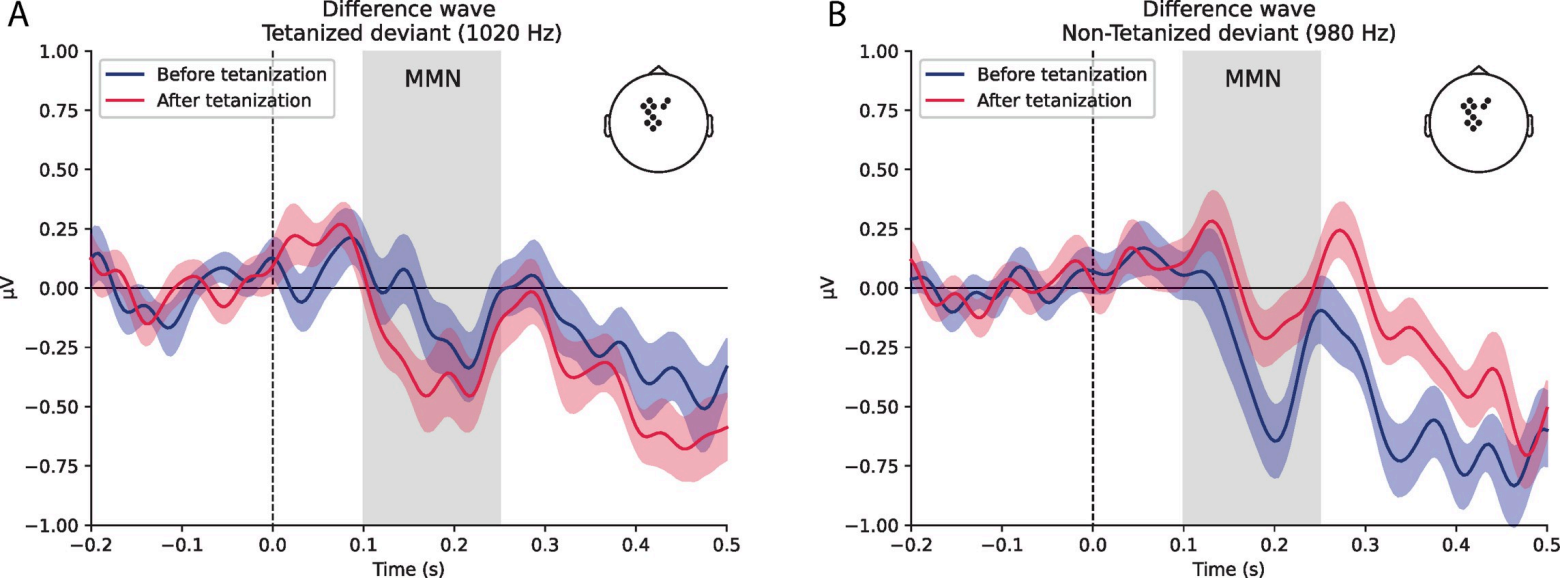
The averaged difference waves (standard minus deviant) for the significant cluster with Tetanization by Stimuli Type interaction A) tetanized deviant (1020 Hz) and B) non-tetanized deviant (980 Hz) before (blue) and after (red) tetanization.

The difference waves (average over cluster in which significant differences were observed) are shown at Fig 3. Deviant stimuli elicit significant negative components at around 200 ms after stimulus–typical latency of mismatch negativity, MMN. The tetanized deviant stimulus has an increased MMN after tetanization, while non-tetanized deviant, in contrast, has a decreased MMN after tetanization.

Noteworthy, the effects of Version by Tetanization, as well as Version by Tetanization by Stimuli Type interaction were insignificant pointing to the negligible effects of this manipulation of the ERP effects of interest.

### Correlation with tones discriminability

Results of psychophysical test revealed that generally participants were able to discriminate the tones as most participants have aprime values above 0.5 (random performance). Nonetheless, there was individual variability in this measure (mean aprime = 0.785+0.13). When all subjects (n = 25) were considered, no changes in discrimination ability can be seen after tetanization for both of tones: t(24) = 0.158, p = 0.875 for tetanized, t(24) = 0.846, p = 0.405 for non-tetanized tones). However, if we excluded all subjects with random performance either before or after tetanization (n = 6) as those who potentially did not pay attention to the task or misunderstood it, there was a trend for increasing the discriminability of the tetanized tones after tetanization, (t(18) = 1.935, p = 0.068). If we do the same for non-tetanized tones of 980 Hz, no changes are seen (t(17) = 0.585, p = 0.566). Thus, while the tetanization effect on behavioral measure is very mild, it seems to alter the discrimination ability of the tetanized tone but did not affect tones of adjacent frequencies.

Next, we examined the correlation of changes in tones discriminability (1020 Hz) with neurophysiological changes after tetanization - MMN in response to 980 and 1020 Hz at a significant fronto-central cluster found at previous stage of analysis.

**Fig 4:**
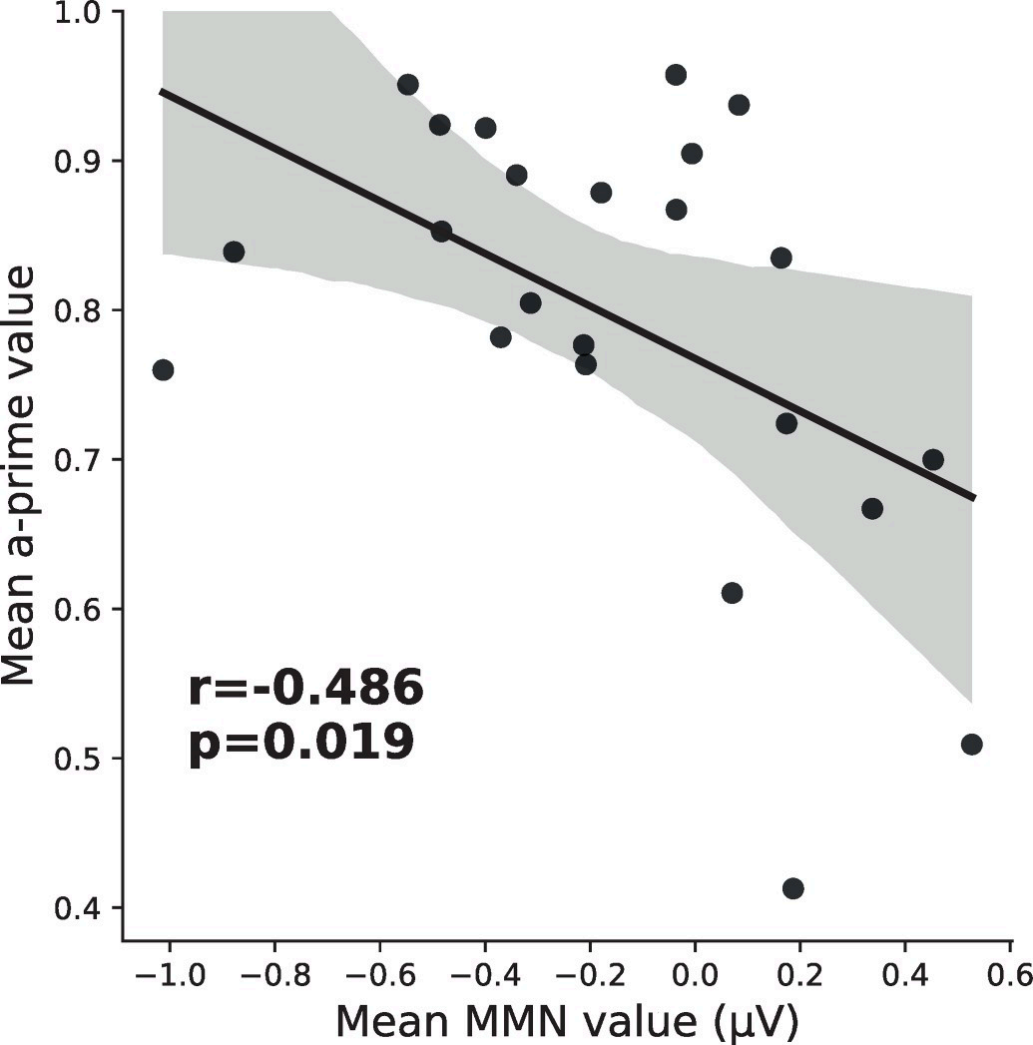
Correlations between individual differences in discriminability of tones and total MMN amplitude for 1020 Hz tone averaged over all conditions.

There was no correlation between the changes in neurophysiological and behavioral response after tetanization ((R(21) = 0.119, p = 0.586) for tetanized tone; (R(21) = -0.184, p = 0.398) for non-tetanized tone), probably because the behavioral effect of tetanization was indeed very small and insignificant. Nevertheless, the total MMN amplitude, averaged over all conditions to increase signal to noise ratio, correlated with individual differences in discriminability of tones: those who were better in discriminating tetanized tones in the psychophysical task had larger MMN response, obtained during passive listening of these tones (R(21) = -0.486; p = 0.013) (Fig 4). Thus, while we could not relate the neurophysiological changes after tetanization to the changes in the sound discrimination ability directly, we confirmed the link of MMN with the ability to discriminate tones reported previously [30, 40].

## Discussion

In the present work we have studied the effect of rapid auditory stimulation on mismatch negativity (MMN) brain response to tones of adjacent frequencies. We have found that MMN, became larger after tetanization in response to tetanized stimuli as compared to MMN to non-tetanized tone of adjacent frequency, thus pointing to better differentiation of these tones in the cortex and generally confirming data by Kompus and Westerhausen [5]. While MMN has been correlated with participants’ discriminability of sounds, our behavioral measures have not been sensitive enough to detect the difference in the tones discriminability after tetanization. In addition to previous study, we have found that sensory tetanization affects not only the response to the tetanized stimuli, but also changes the representation of tones of adjacent frequencies (non-tetanized deviant): its cortical discriminability from other non-tetanized tones (standard stimuli) decreases, which is reflected in the attenuated MMN response for non-tetanized stimuli after tetanization. We believe that this effect is best explained with the reference to the lateral inhibition phenomenon.

Lateral inhibition is the neural mechanism in which an excited neuron inhibits the activity of topologically adjacent neurons via inhibitory interneurons [41]. This mechanism is also essential for learning, as e.g. increased frequency discrimination leads not only to the increased response for the central/best frequency of the neurons but inhibits those in the sidebands, thus, sharpening of the tuning curve of the neurons [42, 43]. In line with these animal studies, musical training has shown to sharpen psychophysical tuning curves in humans [44, 45]. Activation of the auditory cortex by sensory input from the lower levels of the auditory system is also accompanied by inhibition of neurons encoding adjacent frequencies [46] and can cause a decrease in the amplitude of the magnetic counterpart of the N1 component of auditory event-related potential [47]. Apparently, as shown in previous study [29], auditory rapid stimulation also induces similar suppression of non-tetanized tones, as reflected in the selective decrease of the N1 component of auditory event-related potential. Our current study has also confirmed that stimulation with a certain tone, enhancing the activation of the corresponding neurons of the cortex, has a suppressive effect on the cortical representations of neighboring frequencies as assessed by the MMN response.

The MMN in our study has not differed between two types of deviant tones before stimulation, but it became larger to tetanized tone than to non-tetanized after tetanization. This is generally in line with the study of Kompus and Westerhausen [5] that also have shown a significant Tetanization by Stimulus effect. However, Kompus and Westerhausen [5] have reported an MMN increase after tetanization specifically in response to tetanized tone, but have not found an attenuated MMN response for non-tetanized stimuli after tetanization as we do. Such discrepancy might be due to the non optimal experimental settings - usage of nosetip reference electrode (average reference in our study) and the analysis of MMN only in the Fcz electrode (multi-channel cluster analysis in our study). Moreover, if you look at the figures of the Kompus and Westerhausen work, you can see the decrease of MMN to non-tetanized tone after tetanization.

Our study also has confirmed the relationship of MMN with individual differences in the ability to differentiate tones, measured in a separate psychophysical experiment [30, 48]. At the same time, the improvement in tones’ behavioral discriminability after tetanization has been rather small and statistically insignificant. Moreover, the changes in neurophysiological measures after tetanization has not correlated with changes in behavioral measures. However, our task has been very simple, and our measure of discrimination ability has been rude (based on just a few representations of tone pairs). Moreover, as all behavioral testing, tones’ discrimination task required participants’ attention, which might fade after prolonged EEG section. Other tests might be more sensitive to the effect of tetanization at the behavioral level.

Beyond the specificity of the neurophysiological effects of auditory tetanization our study has also supported the other characteristic of long-term potentiation—longevity. While the longest interval between the end of tetanization and test has been 15 minutes, the testing block by itself has lasted for about 15 minutes and stimulus repetitions are needed to obtain the studied ERP components. Thus, the neurophysiological effects of tetanization do not fade immediately after just 2-minutes and lasts for at least about 15 minutes. Whether the effects persist for longer periods of time is above the scope of our study. Corresponding cellular effects in animal studies can persist for several days or even months [49]. For the systemic ERP effect we can only refer to previous work that showed the N1 increase to the tetanized stimulus persisting for one hour [4] or even for 7–35 days [29]. But no MMN effects have been detected in the later study (mind that different stimulation frequency). Thus this question remains for the future research.

Additionally, our study has shown that changing the number of deviants and their probability does not influence the tetanization effect, pointing to the possibility of using a shorter version of the paradigm, which makes it more convenient for testing tetanisation effects in various clinical groups.

Our study has implications in the clinical field as we have shown that non-invasive EEG/ERP study of the auditory tetanization can be used to examine cortical plasticity and effects related to long-term potential (LTP) and lateral inhibition in humans. This paradigm has a potential to be applied in a wide range of clinical settings and population, as no active task is performed and participants can just watch their self-selected videos.

## Conclusions

To sum up, our study presents neurophysiological changes caused by short-term auditory tetanization in humans that are supposed to be linked to the long-term potentiation (LTP) mostly studied at cellular level after electrical stimulation in animals. We have shown that rapid auditory stimulation not only enhances neurophysiological index of tones discriminability (MMN) making it larger for tetanized (1020 Hz) than non-tetanized (980 Hz) sound after stimulation, but also attenuates the MMN in response to non-tetanized sound of adjacent frequency (980 Hz). These effects correspond to the well-known properties of LTP: specificity and longevity (as the effects last for at least 15 minutes), and expands it into higher-level work of the brain, showing the interrelation of sound representations in the cortex and involvement of lateral inhibition in such changes associated with passive learning.
